# Advances of 3D Cell Co-Culture Technology Based on Microfluidic Chips

**DOI:** 10.3390/bios14070336

**Published:** 2024-07-10

**Authors:** Can Li, Wei He, Yihua Song, Xia Zhang, Jianfei Sun, Zuojian Zhou

**Affiliations:** 1 Engineering Research Center of TCM Intelligence Health Service, School of Artificial Intelligence and Information Technology, Nanjing University of Chinese Medicine, Nanjing 210023, China; lican@njucm.edu.cn (C.L.); yihua.song@njucm.edu.cn (Y.S.); zhangxia@njucm.edu.cn (X.Z.); 2 Department of Clinical Medical Engineering, The First Affiliated Hospital of Nanjing Medical University, Nanjing 210029, China; hw66886688@126.com; 3 State Key Laboratory of Bioelectronics and Jiangsu Key Laboratory of Biomaterials and Devices, School of Biological Sciences & Medical Engineering, Southeast University, Nanjing 210009, China

**Keywords:** microchip, co-culture, microfluidic technology, cell cultivation, intercellular communication

## Abstract

Cell co-culture technology aims to study the communication mechanism between cells and to better reveal the interactions and regulatory mechanisms involved in processes such as cell growth, differentiation, apoptosis, and other cellular activities. This is achieved by simulating the complex organismic environment. Such studies are of great significance for understanding the physiological and pathological processes of multicellular organisms. As an emerging cell cultivation technology, 3D cell co-culture technology, based on microfluidic chips, can efficiently, rapidly, and accurately achieve cell co-culture. This is accomplished by leveraging the unique microchannel structures and flow characteristics of microfluidic chips. The technology can simulate the native microenvironment of cell growth, providing a new technical platform for studying intercellular communication. It has been widely used in the research of oncology, immunology, neuroscience, and other fields. In this review, we summarize and provide insights into the design of cell co-culture systems on microfluidic chips, the detection methods employed in co-culture systems, and the applications of these models.

## 1. Introduction

Intercellular communication is a key characteristic of multicellular organisms [1]. Crucially, understanding the communication mechanisms between cells is essential for unveiling the physiological and pathological processes of multicellular organisms, which encompasses cell development and growth, immune interactions, cancer metastasis, cell differentiation, as well as tissue and organ formation [2,3]. Hence, it is necessary to establish a similar in vivo cultivation environment and system to research cell–cell interactions. Cell co-culture technology, based on cell cultivation, has emerged [4]. By co-culturing different types of cells in the same environment, researchers can simulate the in vivo environment to the greatest extent, enabling cells to communicate and support each other for growth and proliferation. Furthermore, this approach allows for the exploration of the mechanisms underlying cell growth, differentiation, apoptosis, and other processes. Additionally, researchers can investigate the mechanism of drug action and targets by detecting the relationships between different cytokines [5]. The cell co-culture system can be applied to many research areas [6], such as cell differentiation, the function and vitality of cells, cell proliferation and migration, metabolic mechanisms, the development of various on-chip organs for drug testing, disease modeling, and personalized medicine.

There are mainly two types of co-culture systems established based on cell co-culture technology. One is the direct co-culture system, which involves physical contact among multiple cells in co-culture; the other is the indirect co-culture system, where co-cultured cells share the same cultivation system without direct contact. The goal of cell co-culture technology is to control parameters like cultivation conditions and cell proportions through the cell co-culture system to promote cell interaction and growth. By constructing a cell co-culture environment, researchers can simulate the complex physiological environment in vivo. It helps researchers to induce cell differentiation, regulate cell proliferation, promote early embryonic development, and provide crucial support and assistance for research in biomedical fields [7]. In native tissues and organs, the microenvironments of cells and their interactions with the surrounding environment are crucial for maintaining the normal structure and function of tissues and organs. In the two-dimensional (2D) cell cultivation method [8,9], cells grow on a flat surface, which cannot simulate the growth state of cells in a 3D environment in vivo, leading to significant differences in cell behavior. This limitation hinders the accurate simulation of the true morphology of cells in in vivo tissues and prevents the achievement of authentic intercellular communication or interaction between cells and the extracellular matrix. Consequently, 2D culture exhibits limited accuracy in drug screening and tissue engineering applications [10]. Moreover, the current understanding of intercellular communication mechanisms is still limited, and intercellular communication in multicellular systems is extremely complex. Traditional 2D cultivation methods fall short of accurately simulating physiological microenvironments and effectively transmitting intercellular communication signals [11,12]. To address these limitations and better simulate the complex intercellular communication found in the real physiological microenvironments, researchers have proposed a three-dimensional (3D) in vitro cultivation method. This method enables cells to grow and differentiate in a 3D space. Compared with the 2D culture method, the 3D culture method [13] promotes cell aggregation and tissue formation through long-term cell cultivation. It also regulates cell morphology, behavior, and function in a 3D physiological environment by manipulating gene and protein expression, proliferation, differentiation, and migration.

Microfluidics technology is a micrometer-scale method designed for processing and manipulating fluids in small channels. This technology operates on scales consistent with mammalian cells, and its unique fluid dynamic manipulation system allows it to mimic a physiological environment more similar to in vivo conditions. Microfluidic technology can co-cultivate multiple cells, generate and control signal gradients, and perform dynamic perfusion cultivation through spatially controllable methods. This enables the precise manipulation of individual cells, the simulation of physiologically relevant microenvironments, and high-throughput analysis under different conditions [14,15,16]. Moreover, it is expected that this technology will contribute to better research on cell growth, cell differentiation, and the effects of drugs on cells, providing a more precise and reliable experimental platform for drug screening and tissue engineering applications. In a 3D microfluidic system, diverse types of cells are cultivated in separate interconnected chambers. Microfluidic devices allow cells to obtain nutrients and oxygen through fluid circulation, exposing them to spatial cues or signal gradients required for cell differentiation, growth, vitality, and proliferation [17,18]. Additionally, microfluidic platforms enable the analysis of dynamic cell–cell interactions under reproducible in vitro cultivation conditions [19]. In recent years, microfluidic systems have been developed and widely used in many areas, such as cancer research [20,21], drug screening [22,23], vascular modeling [24,25], and neuroscience [26], to better evaluate drug efficacy and the feasibility of tissue engineering applications. The cell co-culture technology based on microfluidic chips has become a research hotspot and has been widely applied in fields such as tumor metastasis and analysis [8,27,28], anti-cancer drug screening [29,30,31,32,33], drug absorption, and drug metabolism [29,34,35]. It is expected to be developed into a 3D cultivation model for in vitro physiological research. This article reviews and summarizes the design of cell co-culture system on-chip, the detection of an on-chip cell co-culture system, as well as current applications extended through microfluidic features. And finally, the potential value and future development trends of 3D microfluidic cell culture technology are discussed.

## 2. Design of Cell Co-Culture System On-Chip

**Figure 1:**
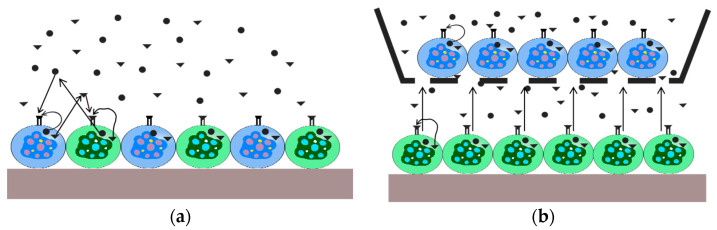
Schematic representation of the interactions in the co-culture systems. (**a**) Direct co-culture system; (**b**) indirect co-culture system.

A microfluidic system is an experimental platform that integrates functions such as actuation, manipulation, monitoring, reaction, detection, and analysis [14]. Microfluidic chips serve as the core components of the microfluidic system [36]. The effective structure contains fluid at the micrometer scale in at least one dimension. Due to its micrometer-scale structure, fluids exhibit specific properties distinct from macroscopic scales [37,38,39]. Additionally, they are compatible with 3D cell cultivation, which opens up new avenues for establishing disease models [17]. The cell co-culture model can be used to observe interactions between cells or cells and their surrounding microenvironment [40,41,42]. Different types of cells can be placed on the same interface [43], and different types of cells can also be placed on separate interfaces to study the effects of certain chemical factors that regulate cell behavior [44]. These two models are named the direct contact co-culture model and indirect contact co-culture model [42,45,46], respectively (see Figure 1). In the direct co-culture model (Figure 1a), communication between cells takes place via direct cell contact, as well as autocrine and paracrine signaling pathways (illustrated by black arrows). On the other hand, in the indirect co-culture system (Figure 1b), there is no physical interaction between cells, and communication occurs solely through autocrine and paracrine mechanisms.

### 2.1. Direct Co-Culture of Cells

The direct contact co-culture of cells on microfluidic chips [47] typically involves the use of droplet technology, trap capture technology, and bioprinting technology to control different cells in the same confined space. Microdroplet technology is a micro technique that utilizes the interaction between flow shear force and surface tension to divide a continuous fluid into discrete microliters/nanoliters or smaller-volume droplets in micrometer-scale channels [48]. During the generation of droplets, two immiscible liquids act as continuous and discrete phases, respectively, and enter different microchannels under the drive of an injection pump with fixed volume flow rate. When the two fluids meet at the intersection, the discrete phase fluid continues to extend to form a “plug like” or “jet like” liquid column. Under the shear and compression of the continuous phase fluid, it fractures due to the instability of the free interface, and the “plug like” or “jet like” liquid column is sandwiched and dispersed in the continuous phase in the form of small-volume units to form droplets. Microdroplets have the characteristics of small volume, large surface area, fast speed, large flow rate, uniform size, closed system, and internal stability. A large number of uniform droplets with picoliter volume can separate the solution to various levels, and individual cells and molecules in each droplet can be visualized, barcoded, and analyzed. By adjusting the geometric structure, two-phase viscosity, flow rate, wettability, and interfacial tension of microfluidic channels, precise control of droplet size, morphology, uniformity, and other parameters can be achieved. Cell capture with microfluidic devices can be divided into contact capture and non-contact capture [49]. Contact capture refers to the direct contact between cells and the trap area during the capture process. This method does not require external energy, but relies on fluid dynamics. On the other hand, non-contact capture refers to the process in which cells do not come into contact with the trap area. However, this method requires external energy, such as electric and magnetic fields. Bioprinting technology can precisely control the arrangement of cells in three-dimensional space and has become the core technology for the fabrication of artificial tissues. Bioprinting works by depositing cells captured in bioink fluids through the use of an automated printhead [50]. In the past few years, microfluidics has been used to enhance droplet-based bioprinting, achieving higher printing accuracy and precision in cell deposition, enhancing the complexity of printed tissues, and promoting new biological applications. The setup with direct contact co-culture of cells allows for direct contact, making it suitable for studying interactions among all cells. The common methods for cell contact include using a shared chamber, where cell suspensions are mixed for culture, or employing a cell-stratified contact culture. In the cell suspension mixed culture approach, more than one cell suspension is directly mixed, and the combined solution is then inoculated into the chip cell culture chamber for co-culture. In the contact culture method, one type of cell is typically aggregated first to form a cell layer. Subsequently, other types of cells are inoculated to adhere to the surface of the initial layer, enabling direct intercellular cross-layer interaction and communication.

**Figure 2:**
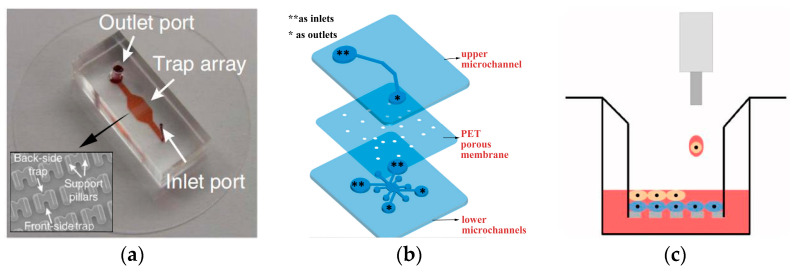
Schematic illustration of the direct co-culture of cell chips. (**a**) Image of the microfluidic cell pairing device with a scanning electron micrograph image of the trap array. Reproduced with permission from [47]. (**b**) Configuration of the multi-channel microfluidic device: exploded view; medium reservoirs are marked with asterisks (** as inlets, * as outlets when loading cells or media). Reproduced with permission from [51]. (**c**) Illustration of the co-culture cell printing platform. Reproduced with permission from [52].

Dura et al. ([47], see Figure 2a) developed a microfluidic device featuring a trap cup array with a scanning electron micrograph image of the trap array for strategically capturing and pairing cells. Each cell trap consists of a single-cell capture cup (the back-side trap) and a double cell capture cup (the front-side trap). The support pillars on each side of the capture cups allow fluid to flow through the cups to guide cells into the traps. This system facilitates the efficient and definitive matching of lymphocytes within a specified duration of interaction, enabling precise evaluation of initial activation occurrences for every pair in controlled microenvironments. Additionally, the platform allows for the simultaneous capture of dynamic processes and static parameters from both partners, facilitating the profiling of lymphocyte interactions over hundreds of pairs in a single experiment with pairwise-correlated multi-parametric analysis. Chen et al. [51] presented a novel microfluidic device with two layers and multiple channels, specifically engineered for the co-culture of cells in direct contact with vessels. The microfluidic device used in the study has one upper microchannel and multiple lower microchannels, separated by a porous membrane of polyethylene terephthalate (PET) with a diameter of 8 µm (Figure 2b). With this apparatus, a co-culture model of the outer blood–retina barrier (oBRB) was developed to replicate the in vivo interaction among retinal pigment epithelial cells, Bruch membrane, and fenestrated choroids. The possibility of evaluating the integrity of the epithelial barrier on a microchip was proven by incorporating platinum electrodes to measure transepithelial electrical resistance (TEER). The design permits the co-culture of cells in direct contact, either between cells or between cells and vessels. Additionally, it can be customized to enable real-time assessment of the state of epithelial monolayers. Dudman et al. [52] employed microvalve technology for bioprinting (as shown in Figure 2c) to generate co-cultures of laminar mesenchymal stromal cells (MSCs) and chondrocytes. The aim was to examine if the addition of MSCs in autologous chondrocyte implantation (ACI) procedures could potentially promote increased synthesis of extracellular matrix (ECM) by chondrocytes. Bioprinting utilizing microvalves employs small-scale solenoid valves (microvalves) to consistently and repeatedly place cells suspended in media. In this research, a laminar co-culture was established by printing MSCs and chondrocytes sequentially into an insert-based transwell system. Revisions were made to the ratios of cell types in order to investigate the capacity of MSCs in promoting ECM production. Histological analysis and indirect immunofluorescence staining demonstrated the formation of dense tissue structures within the chondrocyte and MSC–chondrocyte cell co-cultures, along with the establishment of a proliferative region at the bottom of the tissue. This research presents an innovative approach that facilitates the efficient manufacturing of therapeutically relevant micro-tissue models. These models can be employed in in vitro research for ACI procedure optimization.

The most obvious advantage of the direct contact co-culture method is its ability to demonstrate interactions among cells. For instance, when neural stem cells (NSCs) are co-cultured with microglia, factors secreted by microglia enhance the dopaminergic differentiation of human NSCs [53]. Similarly, co-cultured astrocytes promote neuronal differentiation of NSCs [54]. Moreover, other regulatory factors such as immune cytokines can be added to the co-culture system for cell–cell interaction research. For example, adding interleukin-33 (IL-33) to a mixed culture system containing primary mouse cortical neurons and glial cells revealed that IL-33 induces glial cells to release inflammatory mediators, thereby reducing neuronal mortality in the co-culture system [55].

Additionally, the co-culture system involving feeder cells is also considered a form of direct contact co-culture. In this system, other cells are plated on a monolayer of specific cells, such as granulosa cells, fibroblasts, or fallopian tube epithelial cells [56,57]. These feeder cells are treated with mitotic inhibitors, commonly known as mitomycin, to suppress cell division while preserving their ability to secrete growth factors. The survival and proliferation of other cells depend on the growth factors secreted by the feeder cells. In cell culture processes, the feeder cell layer acts as a promoter of growth and proliferation and an inhibitor of differentiation. This role is particularly important in the cultivation of embryonic stem cells (ESCs) [58,59]. Furthermore, this method often facilitates the formation of cell junctions among multiple cell types. For instance, by extending the co-culture of two cell types, a multicellular system comprising neurons, astrocytes, and microglia has been established. This system more realistically simulates the neuroinflammatory response in the body, providing a better understanding of the impact of cell crosstalk on neuroinflammation [5].

### 2.2. Indirect Co-Culture of Cells

**Figure 3:**
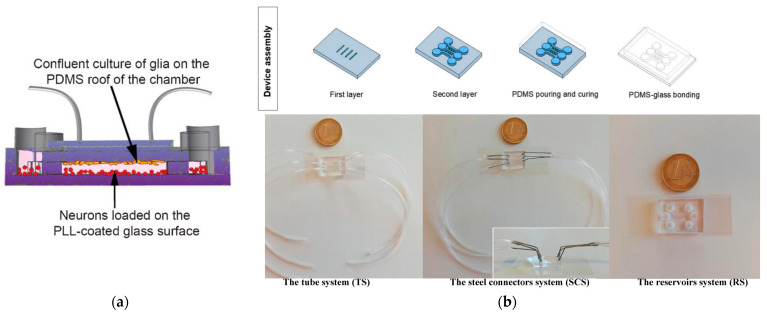
Schematic illustration of the indirect co-culture of cells chips. (**a**) The vertically layered microfluidic platforms with glia (orange spheres) and neuron (red spheres) indirect co-culture. Reproduced with permission from [45]. (**b**) A microfluidic device with multiple compartments for the cultivation of multi-cell populations. Reproduced with permission from [68].

The interaction or regulation among cells can occur not only through direct cell contact but also via chemical signals released in the microenvironment. In the latter case, where direct contact is to be avoided, an indirect co-culture system is required. Indirect contact co-culture of cells involves cultivating multiple cell types through chemical interactions within the culture medium, without direct contact. This method utilizes microvalves, hydrogel, semipermeable membranes, and narrow channels [60,61,62,63] to culture different types of cells in distinct areas of the chip. This indirect co-culture method eliminates the influence of direct cell contact and is suitable for paracrine and endocrine signal transduction research. On microfluidic chips, indirect co-culture of cells can be achieved through both co-chamber [64] and independent chamber [38,39,62,63] setups, mainly realized through microvalve isolation [45,65], channel isolation [38,39,66], and membrane isolation [67]. Microvalve isolation is primarily achieved through a pneumatic drive system that controls the microvalve to form a raised compartment, thereby connecting and isolating chambers [67]. Shi et al. [45] developed a vertically layered setup and a four-chamber setup for co-culturing central nervous system (CNS) neurons and glia (see Figure 3a). The cell compartments in the apparatus were separated by valve barriers that could be activated by pressure, facilitating regulated interaction between the two types of cells. This distinctive design enabled the close co-culturing of glial cells and neurons, the selective transfection of specific neuronal groups, and the real-time observation of neuronal interactions, including the growth of synapses. De et al. (see Figure 3b) reported a microfluidic device with multiple compartments [68], enabling the cultivation of three distinct cell populations in separate fluidic circuits. The chip consists of three perfusable compartments (500 μm wide, either 100 or 250 μm high, and 6 mm long) with distinct inlets and outlets (diameter of 2 mm), interconnected through a series of narrow and parallel microgrooves (either 2.5, 5, or 10 μm wide, 2.5 μm high, and 250 μm long) that can allow the separation between soma and neurites and promote unidirectional neurite elongation from one cell compartment to the adjacent one. The chip setups for cell culture contain the tube system (TS), the steel connector system (SCS) and the reservoir system (RS). The device permits cell migration across the compartments and their differentiation. The researchers showed that optimizing the device’s geometric characteristics and cell culture parameters can enhance the attachment and growth of neuron-like human cells (SH-SY5Y cells), regulate the migration of neurons and Schwann cells between compartments, and facilitate prolonged studies on cell cultures. These discoveries present opportunities for plenty of in vitro co-culture research in neuroscience.

Channel isolation in microfluidic cell co-culture systems can be categorized into independent chamber isolation [38,62,65,68] and shared chamber isolation [39,60]. Currently, the preferred method for studying intercellular communication in microfluidic cell co-culture systems relies on independent chamber isolation. Multiple chambers are isolated using techniques such as microvalves, microarray columns [60], or fluid dynamics [61]. The combination of microfluidic technology and fluid dynamics achieves co-culture of cells in a shared chamber by controlling the liquid flow rate and cell contact with the medium. Various cell co-culture models based on channel isolation have been developed, and research on intercellular communication on microfluidic chips has transitioned from cell population studies to single-cell analyses [69]. Utilizing precise laminar flow control, microfluidic chips with independent chambers and fluid mechanics can automatically and stably move along both sides of the main channel, being introduced into adjacent separation areas to form a non-contact co-culture model. This design is convenient for real-time observations of cell behavior [61,70]. Microfluidic chips combined with shared chambers and fluid dynamics typically consist of two parallel side channels and an intermediate channel [67,71]. The interface of the intermediate channel connects the left and right channels, and the edges of the different compartments are neat and free of cell debris or substances that adversely affect cell migration. Apart from cell population research, shared chamber heart-shaped flute designs [69,70,71,72,73,74] can be used for single-cell capture and analysis. The unique heart-shaped depression design allows effective pairing of different types of cells at the single-cell level, minimizing spatial constraints on cells. By adjusting the number and position of heart-shaped flutes, various functions can be achieved, such as cell pairing ratio control, cell pairing spacing, and the formation of various single-cell arrays.

Using porous membrane isolation, the two channels are connected by substrate membranes of different materials, such as polycarbonate membrane, polyethylene (PE) membrane, etc. In this setup, one type of cell is cultured at the bottom of the chip, while the other type of cell is cultured at the top of the membrane [75]. Alternatively, the microfluidic characteristics can be leveraged to inoculate cells on both sides of the membrane [76,77]. The layered structure on the chip supports long-term co-culture of cells. However, the single-channel layered co-culture model based on membrane isolation faces challenges in maintaining long-term stable stratification among cells. Over time, it may evolve into a random co-culture monolayer. Cells often tend to aggregate at the entrance and exit of microchannels, limiting its application in more physiologically relevant research. On the other hand, the dual-channel co-culture model based on membrane isolation addresses these issues by increasing the channel height, resulting in a more uniform cell layer and stable cell morphology.

In general, the direct co-culture method of cells is simple, usually requiring the placement of two or more types of cells in the same culture dish in specific proportions for direct interaction between cells. Due to the direct interaction between cells, this method can better retain the connection information between cells and make the cultured cells more similar to their in vivo state. It is suitable for studying the interaction between adjacent tissue cells, cellular interactions, and the induction of cell differentiation. However, due to the direct interaction between cells, separating the two types of cells becomes more difficult, making observation and subsequent detection inconvenient. Additionally, during direct co-culture, different cells may influence each other, affecting the analysis of experimental results. In indirect co-culture, cells do not directly interact but communicate through cell-secreted factors, facilitating subsequent cell separation and detection. Indirect co-culture can be used for studies on cytokine-induced cell differentiation and proliferation, simulating the liquid circulation environment in the body, and studying intracellular (autocrine) and intercellular (paracrine) interactions. However, due to the lack of direct contact, direct interactions between cells may not be observed. Special culture plates and chambers are required, which can be costly. Using microfluidic cell chips requires relatively complex operations, high technical expertise, and specialized equipment.

## 3. Detection of Cell Co-Culture System by Microfluidics

After constructing an on-chip cell co-culture system, it is crucial to evaluate the system or barrier to determine its suitability for studying intercellular communication mechanisms. This evaluation involves assessing both system or barrier permeability and intercellular interactions, as illustrated in Table 1.

In existing research, the assessment of system or barrier permeability primarily involves detecting the permeability of molecules, evaluating cell viability, measuring electrophysiological activity, identifying cell markers, and observing cell morphogenesis using electron microscopy. Detection of permeability molecules, such as glucose [78] and rhodamine [79], enables the assessment of the hydrogel penetrability within the system. Cell viability is determined through methods like trypan blue colorimetry/thiazolyl blue colorimetry [80,81], live or dead cell staining [82], and lactic dehydrogenase (LDH) activity assay [83,84], providing insights into whether the system offers a conducive environment for cell co-culture. Electrophysiological activity detection, often applied to neural cells and neurons, utilizes techniques like patch clamp technology [61] and transmembrane resistance (TEER) [85,86,87] to measure and quantitatively evaluate barrier permeability. Cell markers [45,88,89,90] and cell morphology [91,92,93] are utilized for detecting the formation of a co-cultured 3D culture pattern. After verifying the feasibility of the cell co-culture system, the simulation of the native microenvironment enables further research on intercellular interactions. This includes studying cell migration [80,81,89,90,94], cell differentiation [95,96,97,98], cell fibrosis [70,99,100,101], and cell toxicity testing [102,103]. On-chip cell migration refers to movement influenced by signals from other cells, involving processes like angiogenesis and cancer metastasis. Migration distance is mainly observed through microscopy. Cell differentiation and fibrosis are marked by specific cells using immunofluorescence staining methods [45], and confocal microscopy is employed to observe and analyze the selective expression of cells in time and space, including changes in cell morphology and group dynamics. Toxicity testing involves detecting changes in signaling factors related to interactions, allowing the study of the impact of targeted drugs on the system. Jeong [104] analyzed the migration ability of fibroblasts towards the 3D tumor chamber by measuring the migration distance of the nucleus of fibroblasts in the culture medium channel. In this co-culture system, the sensitivity differences of paclitaxel drug therapy were studied by comparing the changes in the proportion of live and dead cells.

After building the on-chip cell co-culture system, it is crucial to evaluate the system’s applicability or barrier function to ensure the accuracy and reliability of experimental results. During the evaluation process, key points such as cell growth and differentiation, intercellular interactions, simulation of the internal environment, barrier function, stability, and repeatability need to be focused on.

## 4. Application

In on-chip cell co-culture models, the most common studied system is the vascular system, followed by blood–brain barrier chips, gas–blood barrier chips, and other organoid chips. Let us explore the applications of microfluidic cell co-culture technology in angiogenesis chips, blood–brain barrier chips, gas–blood barrier chips, and organoid chips.

### 4.1. Angiogenesis

Angiogenesis chips are typically designed with microgaps, often combined with a 3D gel to serve as a scaffold for angiogenesis. This chip structure facilitates cell connectivity, promotes the formation of vascular networks, and creates a reliable three-dimensional microenvironment. Such designs offer valuable insights for clinical medicine and tissue engineering. In cardiovascular model establishment, simulating and studying fluid stress often involves inserting a membrane to replicate the three-dimensional cardiovascular environment. Sometimes, the inserted membrane becomes a key component for studying the biological mechanisms of blood vessels and heart valves. Co-culture systems on these chips have also been used to study the impact of certain types of cancer cells on angiogenesis [105,106,107]. Such studies have revealed a close relationship between vascular networks and the occurrence and development of tumors [108].

**Figure 4:**
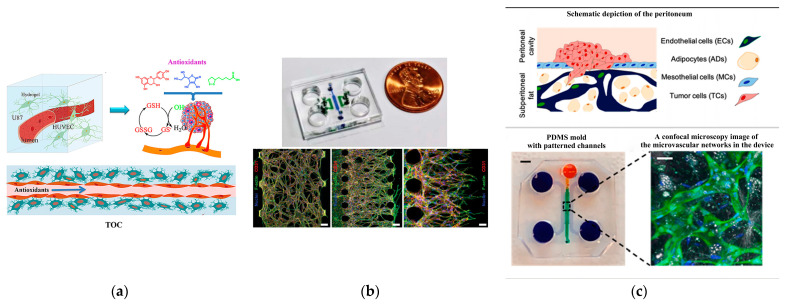
Angiogenesis chips. (**a**) The three-dimensional tumor microvascular structure simulated on a microfluidic chip for study of antioxidant effects on malignant glioma cells in vitro. Reproduced with permission from [79]. (**b**) Microfluidic chip design and confocal micrographs showing the overall architectures of vascular networks established by vasculogenic and angiogenic processes at day 4 (scale bars, 100 mm), as well as angiogenic sprouts grown for 2 days (scale bar, 50 mm). Reproduced with permission from [109]. (**c**) In vitro microvascular network model of the peritoneum produced with polydimethylsiloxane (PDMS) employing the technique of soft lithography. Reproduced with permission from [105].

Liu et al. [79] conducted a study using a microfluidic chip to simulate the three-dimensional tumor microvascular structure and investigated the impact of antioxidants on malignant glioma cells in vitro. They utilized hydrogel to construct a 3D chamber for co-culturing endothelial cells and glioma cells, creating a simulated environment for tumor microvasculature (see Figure 4a). A macroporous gelatin transglutaminase hydrogel with favorable biomechanical properties for cell culture and nutrient renewal was employed in the study. In another investigation, Kim et al. [109] (see Figure 4b) observed that the angiogenesis of human umbilical vein endothelial cells (HUVECs) relies on co-culturing with human lung fibroblasts (LFs), as the formation of an interconnected vascular network was not observed in the HUVEC system not co-cultured with LFs. Ibrahim et al. [105] investigated the influence of stromal cell effects on the attachment and proliferation of tumor cells, along with the reciprocal consequences of tumor cells on vascular and mesothelial permeability. They employed an in vitro model of the vascularized human peritoneal omentum and ovarian tumor microenvironment (TME) to investigate metastases at both early and advanced stages. The results indicated that the growth of tumors resulted in a reduction in microvascular permeability through physical mechanisms, while simultaneously inducing an elevation in microvascular permeability via cytokine signaling. This emphasizes the sophistication and potential conflicting roles of tumor cells in the development of ascites. The developed system functions as a sturdy platform for investigating the interactions between tumor cells and stromal cells during the spread of ovarian cancer within the peritoneal cavity, presenting a novel in vitro vascularized model of the human peritoneum and ovarian cancer TME, which is shown in Figure 4c.

### 4.2. Blood–Brain Barrier

**Figure 5:**
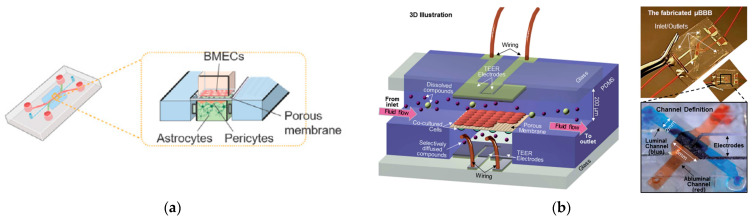
Blood–brain barrier (BBB)-on-a-chip. (**a**) Microfluidic BBB-on-a-chip with the cross-sectional view. Reproduced with permission from [110]. (**b**) Structure and design of the multi-layered microfluidic device (μBBB). Reproduced with permission from [114].

The blood–brain barrier is a dense barrier structure composed of vascular endothelial cells, astrocytes, pericytes, and basement membrane in the brain, playing a crucial role in maintaining the stability of the central nervous system environment [110,111]. The most common co-culture model for the nervous system and blood–brain barrier involves astrocytes and endothelial cells, with chip designs often incorporating microchannel connections to simulate axonal guidance function [37,112,113]. Microfluidic structures for the blood–brain barrier on the chip come in two main types: planar and vertical. The planar model typically utilizes microcolumns or microchannel arrays as the boundary between the blood and lateral brain cavities (such as Figure 5a). These microstructures have gaps small enough to capture cells on either side, enabling co-culturing of both cell types on their respective sides, similar to vertically placed porous membranes.

The vertical model utilizes a porous membrane as the boundary between blood vessels and brain tissue ([114], see Figure 5b). In Figure 5b, the top channel contains brain microvascular endothelial cells (BMECs) and the bottom channel contains astrocytes and a media pool. A porous membrane is sandwiched in between these two channels. Positioned between these two microfluidic structures, one side accommodates blood vessels, while the other supports brain tissue. Chip designs for the blood–brain barrier often incorporate microchannel connections due to their ability to simulate axonal guidance functions. To establish an optimal in vitro model of the blood–brain barrier, it is crucial to replicate key physical characteristics of the cerebral capillary microenvironment including fluid flow, extracellular matrix, and the cylindrical geometric structure of normal brain microvessels. Astrocytes play a vital role in promoting the formation of the blood–brain barrier, assisting in the maturation of neurovascular endothelial cells, and facilitating a tighter connection for barrier integrity. Jeong et al. [83] introduced a multi-chamber microfluidic blood–brain barrier chip designed to recapitulate the key functions of the blood–brain barrier at the astrocyte capillary interface. The chip optimizes physiological shear stress and extracellular matrix conditions to enhance the formation of tight cell junctions. Notably, this design allows for the simultaneous conduct of up to 16 different tests on a single chip.

### 4.3. Blood–Gas Barrier

**Figure 6:**
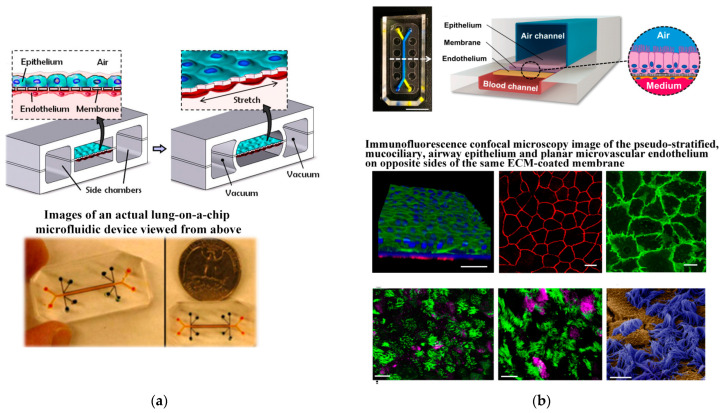
The design of blood–gas barrier by microdevice. (**a**) The microfabricated lung mimic device utilizes compartmentalized PDMS microchannels to form an alveolar–capillary barrier on a thin porous flexible PDMS membrane coated with ECM. Reproduced with permission from [117]. (**b**) The human small airway-on-a-chip. Reproduced with permission from [118].

The blood–gas barrier is a complex tissue structure crucial for facilitating normal gas exchange in the body. It comprises the liquid layer on the surface of alveoli, type I alveolar cells, basement membrane, a thin layer of connective tissue, capillary basement membrane, and endothelium of pulmonary capillaries [115]. The formation of the blood–gas barrier is intricately linked to the construction of the alveolar–capillary interface. To recreate the connection between alveoli and blood vessels, alveolar cells and vascular endothelial cells are co-cultured, and the essential function of alveolar capillaries is simulated through gas–liquid exposure [116]. The membrane-based separation channel structure allows independent manipulation of fluid flow, as well as the transfer of cells and nutrients. Additionally, lung function involves cell stretching during respiratory movement, necessitating the application of mechanical forces on the chip to mimic the dynamic mechanical deformation of the alveolar–capillary interface caused by respiratory movement. Huh et al. [117] constructed a chip model of alveolar pulmonary capillary units (see Figure 6a), successfully replicating the dynamic mechanical deformation of the alveolar–capillary interface caused by respiratory movement. Human alveolar epithelial cells and human pulmonary microvascular endothelial cells are cultured on both sides of the membrane. As cells form layers, air is introduced into the epithelial compartment, creating a gas–liquid interface that more accurately simulates the inner layer of alveolar air space. To model the human small airway-on-a-chip, Benam et al. ([118]) used soft lithography to create a microfluidic device made of PDMS containing an upper channel with a height and width (both 1 mm) similar to the radius of a human bronchiole separated from a parallel lower microvascular channel (0.2 mm high, 1 mm wide) by a thin, porous (0.4 µm), polyester membrane coated on both sides with type I collagen (see Figure 6b). The primary human airway epithelial cells (hAECs) isolated from healthy donors or COPD patients were cultured on top of the membrane until confluent with medium flowing (60 µL/h) in both channels. To trigger lung airway epithelial differentiation, the apical medium was removed after five days and air was introduced to create an air–liquid interface (ALI), while retinoic acid (3 µg/ mL) was added to the medium flowing in the lower channel to prevent the development of a squamous phenotype. Three to five weeks later, primary human lung microvascular endothelial cells were seeded on the opposite side of the porous membrane and cultured at the same flow rate until confluent to create a tissue–tissue interface. Immunofluorescence confocal microscopic analysis revealed that these culture conditions resulted in the formation of a pseudo-stratified, mucociliary, airway epithelium and a planar microvascular endothelium on opposite sides of the same ECM-coated membrane.

### 4.4. Other Organoid Chips

The organoid chip is an innovative biomedical technology designed to replicate the physiological processes of various organs in the human body by cultivating human tissues or organ cells on the chip. This approach offers a more realistic, rapid, and accurate model for disease treatment and the development of new drugs [23,30,118,119,120]. Initially proposed by Sin et al. [121], the organoid chip aims to construct and simulate the microenvironment of different tissues and organs in the human body, with continuous advancements and developments. In addition to the organoid chips mentioned earlier, recent progress has expanded the repertoire to include kidney chips [122], liver chips [123], intestinal chips [124,125], bone chips [126], tumor chips [127], and multi-organ chip [128], broadening the applications of these human organoid chips.

**Figure 7:**
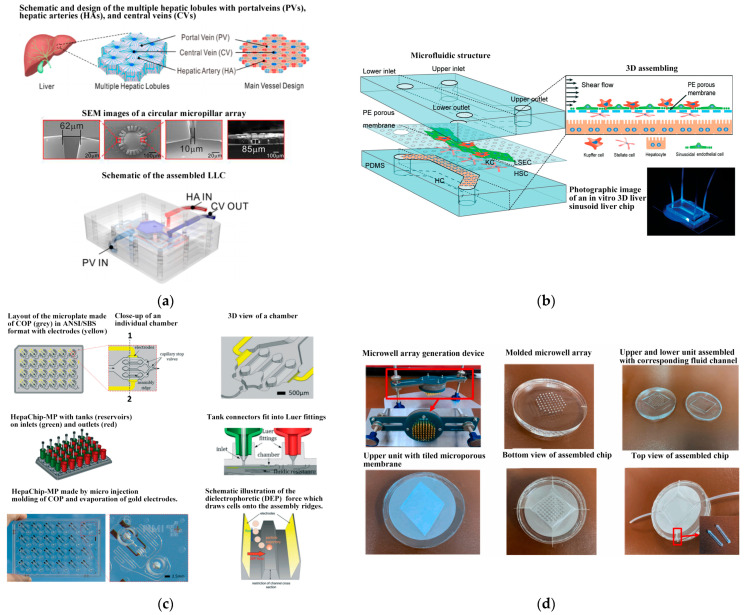
Schematic components of the liver lobule chip (LLC). (**a**) Schematic, design, and characterization of the multiple hepatic lobules with portalveins (PVs), hepatic arteries (HAs), and central veins (CVs). Reproduced with permission from [130], (**b**) Schematic of the in vitro 3D liver sinusoid liver chip. Reproduced with permission from [132]. (**c**) The HepaChip-MP design. Reproduced with permission from [133]. (**d**) The 3D-DMLoC system. Reproduced with permission from [134].

The liver sinusoid wave chip is a common model for the liver, primarily utilizing the co-culture of liver cells and endothelial cells to simulate the structure of liver sinusoids [129]. The microfluidic structure of the liver model on the chip mainly involves membrane-isolated indirect co-culture. This model can be extended to various liver biology research and liver-related disease studies, such as drug-induced liver toxicology, cancer research, and the pathological effects of various hepatophilic infectious factors. Research has indicated that the flow of mediators in liver cell secretions and the dynamic interaction with collagen play a crucial role in maintaining primary liver cell function [129,130,131]. Therefore, liver cells were cultured on a microfluidic platform under the condition of flow perfusion of the culture medium and covered with collagen to investigate the interaction between medium flow, collagen production, and liver cell function. Ya et al. ([130], see Figure 7a) developed a lifelike bionic liver lobule chip (LLC), on which perfusable hepatic sinusoid networks were achieved using a microflow-guided angiogenesis methodology. To accurately replicate the structure of liver sinusoids, Du et al. ([132], see Figure 7b) developed an in vitro three-dimensional liver chip comprising four primary liver cell types under shear flow, aiming to mimic the liver microenvironment with precise cell composition and quantified physical interactions. Busche et al. ([133], see Figure 7c) established a novel, parallelized, and scalable microfluidic in vitro liver model demonstrating hepatocyte function. This model showcased fully automated cell culture preparation in the HepaChip microplate (HepaChip-MP) using a pipetting robot. The HepaChip-MP consists of 24 independent culture chambers. An automated dielectrophoresis process selectively assembles viable cells into elongated microtissues. Freshly isolated primary human hepatocytes (PHHs) and primary human liver endothelial cells (HuLECs) were successfully assembled as co-cultures, mimicking the liver sinusoid. The establishment of microtissues using the HepaChip-MP necessitates only small amounts of primary human cells. The system is expected to be integrated into routine procedures in cell culture labs, enabling comprehensive investigations on liver biology and its potential applications in preclinical drug development. Zheng et al. [134] developed a 3D dynamic multi-cellular liver-on-a-chip device (3D-DMLoC) to replicate the microenvironment of liver tissue in vivo (see Figure 7d). The device incorporated functionalities like simulated hepatic sinusoid, perisinusoidal space, and continuous liquid perfusion, resulting in the formation of 3D cell spheroids. The HepaRG cells and HUVECs were co-cultured for 7 days within this chip. The observed liver toxicity was correlated with acute hepatocyte injury, which was indicated by the ratios of secreted AST/ALT contents. The liver-on-a-chip device demonstrated successful development and validation, providing a more accurate reproduction of the in vivo physiological microenvironment of the liver. This platform holds promise for the easy, efficient, and accurate screening of potential hepatotoxic chemicals in the future.

**Figure 8:**
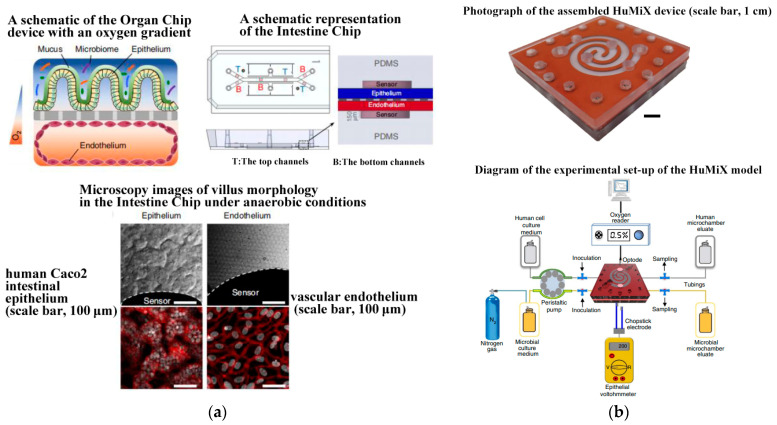
Gut chips. (**a**) Oxygen-sensitive human intestine chip microfluidic culture device. Reproduced with permission from [124]. (**b**) The in vitro model (HuMiX) of the gastrointestinal human–microbe interface. Reproduced with permission from [135].

**Figure 9:**
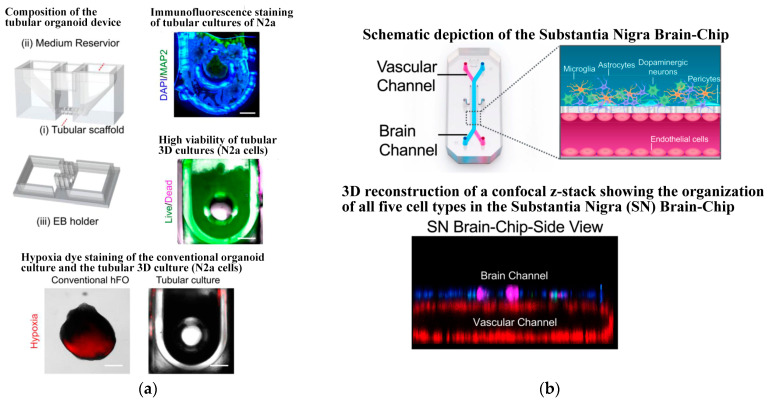
Brain chips based on microfluidics. (**a**) Tubular organoid device design and validation. Reproduced with permission from [131]. (**b**) The human substantia nigra brain chip. Reproduced with permission from [136].

**Figure 10:**
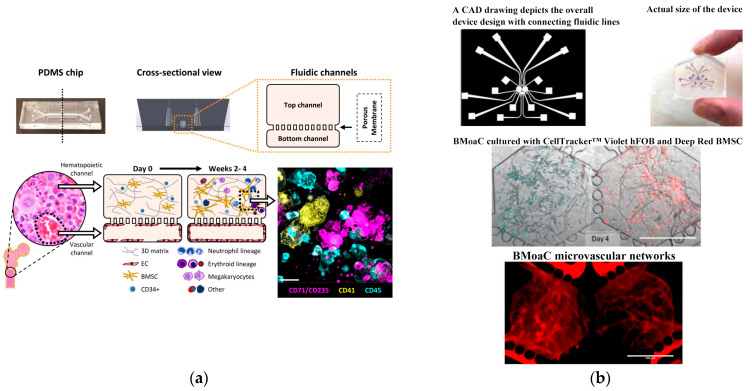
Bone chips based on microfluidics. (**a**) Primary human bone marrow chip supports in vitro hematopoiesis over 4 weeks in culture and improves CD34+ progenitor survival and colony forming capacity. Reproduced with permission from [137]. (**b**) Vascularized bone marrow chip. Reproduced with permission from [126].

Since 2019, Jalili-Firoozinezhad et al. [124] and Puschhof et al. [125] have explored the development of intestinal chips, emphasizing the interaction between the gut and microorganisms. The former employed stretchable materials to replicate the rhythmic peristalsis and contraction of the intestine, as depicted in Figure 8a. The latter focused on the interaction between intestinal epithelium and microorganisms to investigate the influence of gut microbiota on health and disease. Shah et al. [135] presented a modular, microfluidics-based model (HuMiX, human–microbial crosstalk), which allows co-culture of human and microbial cells under conditions representative of the gastrointestinal human–microbe interface. The model integrates oxygen sensors (optodes) for the real-time monitoring of the dissolved oxygen concentrations within the device. In addition, they also fabricated a specially designed version of HuMiX, which allows the insertion of a commercial chopstick-style electrode to monitor TEER for the characterization of cell growth and differentiation within the device (see Figure 8b). In 2021, Ao et al. [131] engineered a brain chip (see Figure 9a) by co-culturing glial cells and immune cells, aiming to simulate the tissue structure and function and offering novel research perspectives for brain diseases. Pediaditakis et al. ([136], see Figure 9b) leveraged the Organs-on-Chips technology to develop a human brain chip representative of the substantia nigra area of the brain containing dopaminergic neurons, astrocytes, microglia, pericytes, and microvascular brain endothelial cells, cultured under fluid flow. The αSyn fibril-induced model was capable of reproducing several key aspects of Parkinson’s disease, including accumulation of phosphorylated αSyn (pSer129-αSyn), mitochondrial impairment, neuroinflammation, and compromised barrier function. This model may enable research into the dynamics of cell–cell interactions in human synucleinopathies and serve as a testing platform for target identification and validation of novel therapeutics. Chou et al. [137] showed a vascularized human bone-marrow-on-a-chip (see Figure 10a) that supports the differentiation and maturation of multiple blood cell lineages over 4 weeks while improving CD34+ cell maintenance, and it recapitulates aspects of marrow injury, including myeloerythroid toxicity after clinically relevant exposures to chemotherapeutic drugs and ionizing radiation as well as marrow recovery after drug-induced myelosuppression. The chip comprises a fluidic channel filled with a fibrin gel in which CD34+ cells and bone-marrow-derived stromal cells are co-cultured, a parallel channel lined by human vascular endothelium and perfused with culture medium, and a porous membrane separating the two channels. As an in vitro model of hematopoietic dysfunction, the bone-marrow-on-a-chip may serve as a human-specific alternative to animal testing for the study of bone marrow pathophysiology. Subsequently, in 2022, Glaser et al. constructed a vascularized bone marrow chip [126], illustrated in Figure 10b. They established an in vitro simulated vascularized bone marrow microenvironment using microfluidics and stem cell technology. This model features a dynamic and permeable vascular network, faithfully reproducing bone marrow function and providing a novel platform for understanding human bone marrow function and the mechanisms of action of related drugs.

**Figure 11:**
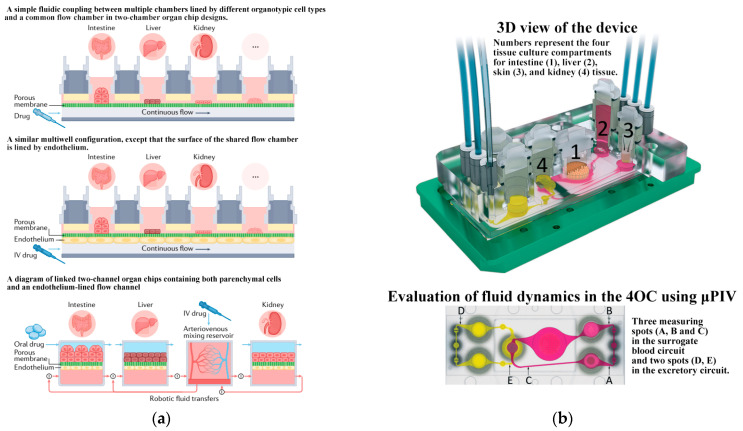
Human chips. (**a**) Schematics showing different multi-organ human body-on-chip formats. Reproduced with permission from [138]. (**b**) The microfluidic four-organ chip device. Reproduced with permission from [139].

A single-organ chip can replicate the physiological and pathological processes of a specific organ in the human body. However, a true biological system emerges from the interaction of multiple organs. Therefore, the development of multiple-organ chips is considered a crucial direction for future advancements. In recent years, scientists have created various organ chips combined to form human chips (Human-on-a-Chip) [132,133,136] ([138], see Figure 11a). Maschmeyer et al. [139] first introduced a microphysiological four-organ chip system that enables a reproducible 28-day co-culture of four tissues (intestine, liver, skin, and kidney tissue). Barrier integrity, continuous molecular transport against gradients, and metabolic activity could be demonstrated for the four-organ chip (4OC) co-cultures, thus making it a perfect platform for further in vitro ADME (absorption, distribution, metabolism, and excretion) and repeated dose toxicity testing. It enhances the simulation of interactions and complex physiological and pathological processes between multiple organs in the human body, thereby improving the efficiency and accuracy of drug screening and evaluation. Table 2 summarizes the typical applications of microfluidic chips and their co-cultured cell/organ types in this review.

Organ chips are extensively applied in drug screening and toxicity assessment [140,141]. In drug development, these chips provide a rapid and accurate method to evaluate drug toxicity and efficacy, offering a more reliable and efficient screening approach for new drug development. Concerning toxicity assessment, organ chips better emulate the human body’s responses to various chemical substances, enhancing the precision and efficiency of drug toxicity evaluation. This contributes to a more scientific and reliable method for safety assessments of chemical substances. Moreover, artificial intelligence technology [142] is employed to design and optimize the structure and function of organ chips, improving their simulation effectiveness and accuracy. This technology allows precise regulation of cell activity in organ chips, enabling advancements in drug screening, toxicity testing, and research on disease pathogenesis [143]. As human chip technology continues to develop, organ chips will increasingly play a vital role in fields such as life sciences, medicine, and drug research.

## 5. Conclusions

Cell co-culture technology based on microfluidic chips can replicate the native microenvironment, capturing the complexity of metabolism and regulation. This technology serves as a novel platform for studying cell–cell communication and holds significant value in uncovering the physiological and pathological processes of multicellular organisms. Currently, the design of cell co-culture systems on microfluidic chips predominantly involves two approaches: direct contact co-culture and indirect contact co-culture. The direct co-culture method typically adopts a co-chamber design, while the indirect contact co-culture employs both co-chambers and independent chambers that utilize microvalve isolation, channel isolation, and membrane isolation. After constructing a cell co-culture system on the chip, the functionality of the co-culture system or barriers can be evaluated through various methods, including permeability molecule detection, cell viability assessment, electrophysiological activity detection, cell marker detection, and electron microscopy observation of cell morphology. These evaluations help determine the system’s suitability for studying intercellular communication mechanisms. Once the feasibility of the co-culture system is confirmed, it becomes a valuable tool for simulating the native microenvironment and conducting subsequent research on cell communication mechanisms. This includes investigating processes such as cell migration, differentiation, fibrosis, and toxicity detection. With advancements in microfluidic cell co-culture technology, diverse chip models have been constructed. Notably, the vascular system has been the most modeled tissue, closely followed by the blood–brain barrier, blood–gas barrier, and other organoid models. The establishment of co-culture models on microfluidic chips creates structures similar to in vivo tissues or organs, addressing limitations of traditional two-dimensional cell culture. These models find applications in basic in vitro research and various fields such as targeted drug screening and toxicity detection.

As technology progresses, on-chip cell co culture technology is evolving from simple multicellular models towards organoids. This shift aims to fully simulate organ-level functions necessary for physiological homeostasis and complex disease processes. However, organoid models are the latest technology in human tissue experimental research. Compared to traditional models, they are still in the exploratory stage. The stability, repeatability, scalability, and precise control of microenvironmental conditions have become issues that need to be overcome in the development of organoid co-culture technology. To create relevant co-culture systems for cell interaction research, it is necessary to integrate organoid models with standardized microdevices. Looking ahead, the development of the organoid chip model paves the way for constructing a human system on-chip through fluid connections. This advanced model has the potential to simulate interactions and physiological reactions of multiple organs at the system level, proving effective in diverse fields including medicine, life sciences, and environmental sciences.

## Figures and Tables

**Table 1:** Detection of co-culture system.

Detection Classification	Detection Target	Detection Method or Marking	Ref.
System or barrier permeability assessment	The permeability of molecules	Glucose	[78]
		Rhodamine	[79]
	Cell viability	Colorimetry	[80,81]
		Staining of live/dead cells	[82]
		Lactic dehydrogenase (LDH) Activity assay	[83,84]
	Electrophysiological activity	Transendothelial electrical resistance (TEER)	[85,86,87]
	Immunofluorescence of cellular marker substances	Actin	[88]
		Green fluorescence protein (GFP)	[45,89,90]
	Formation of spherical bodies	Electron microscope	[91,92,93]
Intercellular interaction	Cell migration	Mass spectrometry analysis, qPCR, Immunofluorescence	[80,81,88,89,94]
	Cell differentiation		[95,96,97,98]
	Cellular fibrosis		[70,99,100,101]
	Cytotoxicity testing		[102,103]

**Table 2:** Application of microchips.

Application		Co-Culture Type	Ref.
Angiogenesis		Endothelial cells, Glioma cells	[79]
		HUVECs, LFs	[109]
		Endothelial cells, Adipocytes, Mesothelial cells, Tumor cells	[105]
Blood–brain Barrier		Astrocytes, Endothelial cells	[110,114]
Blood–gas Barrier		Human alveolar epithelial cells, Human pulmonary microvascular endothelial cells	[117]
		HAECs, primary human lung microvascular endothelial cells	[118]
Other Organoid Chips	liver chips	liver cells, endothelial cells	[130,132,133,134]
	intestinal chips	intestinal epithelium, microorganisms	[125,135]
	bone chips	CD34+cells, bone-marrow-derived stromal cells, human vascular endothelium	[137]
	brain chips	glial cells and immune cells	[131]
		dopaminergic neurons, astrocytes, microglia, pericytes, and microvascular brain endothelial cells	[136]
	multi-organ chip	intestine, liver, skin, and kidney tissue	[139]

## Data Availability

Not applicable.
